# Development of a prediction nomogram for IgG levels among asymptomatic or mild patients with COVID-19

**DOI:** 10.3389/fcimb.2024.1477585

**Published:** 2024-12-09

**Authors:** Jianying Yi, Zhili Liu, Xi Cao, Lili Pi, Chunlei Zhou, Hong Mu

**Affiliations:** ^1^ Department of Clinical Laboratory, Tianjin First Central Hospital, School of Medicine, Nankai University, Tianjin, China; ^2^ Department of Clinical Laboratory, The Third Central Hospital, Tianjin, China; ^3^ Tianjin Key Laboratory of Extracorporeal Life Support for Critical Diseases, Tianjin, China; ^4^ Artificial Cell Engineering Technology Research Center, Tianjin, China; ^5^ Tianjin Institute of Hepatobiliary Disease, Tianjin, China

**Keywords:** SARS-CoV-2, long COVID, IgG, IL-6, vaccine, comorbidity

## Abstract

**Objective:**

COVID-19 has evolved into a seasonal coronavirus disease, characterized by prolonged infection duration and repeated infections, significantly increasing the risk of patients developing long COVID. Our research focused on the immune responses in asymptomatic and mild cases, particularly the critical factors influencing serum immunoglobulin G (IgG) levels and their predictive value.

**Methods:**

We conducted a retrospective analysis on data from 1939 asymptomatic or mildly symptomatic COVID-19 patients hospitalized between September 2022 and June 2023. Spearman methods were used to test the correlation between serum IgG and age, immunoglobulin M (IgM), procalcitonin (PCT), interleukin-6 (IL-6), nucleic acid conversion time, and BMI. Univariate and multivariate logistic regression analyses identified independent key factors influencing serum IgG levels, which were integrated and visualized in a nomogram. Finally, receiver operating characteristic (ROC) curves were plotted to predict the model’s diagnostic performance by calculating the AUC.

**Results:**

Mild patients showed higher levels of IgG, IgM, and longer nucleic acid conversion times than asymptomatic patients, and a lower proportion of them had received ≥ 3 COVID-19 vaccine doses. Serum IgG was positively correlated with serum IgM and negatively correlated with age, PCT, IL-6, and BMI. Notably, it showed a moderate negative correlation with nucleic acid conversion time (r = -0.578, *P* < 0.001). Logistic regression results showed that younger age, lower IL-6 levels, ≥ 3 doses of vaccine, and no comorbidities were independent predictors of serum IgG levels ≥ 21.08 g/L. We used age, IL-6 levels, vaccine doses, and comorbidities to create a nomogram for predicting serum IgG levels, with the area under the ROC curve reaching 0.772.

**Conclusion:**

Age, IL-6 levels, vaccination status, and comorbidities were independent predictors of serum IgG levels in asymptomatic or mild COVID-19 patients, facilitating risk stratification and clinical decision-making. Notably, receiving ≥3 doses of the COVID-19 vaccine was the most beneficial factor for elevated serum IgG levels.

## Introduction

Since it first emerged in Wuhan in December 2019, COVID-19, caused by severe acute respiratory syndrome coronavirus 2 (SARS-CoV-2), has coexisted with humans globally for more than four years. With the more transmissible Omicron becoming the dominant strain, its virulence significantly decreased. Combined with widespread vaccination against SARS-CoV-2, most COVID-19 infections are now asymptomatic or mild, with severe cases and mortality rates significantly reduced (Hall et al., 2022; Soheili et al., 2023; Trobajo-Sanmartin et al., 2023). Current evidence indicates that about 10-20% of individuals, after recovering from the initial illness, suffer from long COVID, which includes recurrent fatigue, shortness of breath, muscle weakness, sleep disturbances, anxiety, and depression, resulting in a diminished quality of life (Soriano et al., 2022; Lopez-Leon et al., 2021; Huang et al., 2023). Consequently, focus has shifted to interventions aimed at alleviating long COVID, especially those that support immune function and reduce inflammation.

It is well known that IgM is produced when the human body first encounters SARS-CoV-2, indicating an active COVID-19 infection. In contrast, serum IgG serves as a protective antibody, signaling either recovery from or past exposure to the infection. IgG binds to the receptor-binding domain of SARS-CoV-2, such as the primary receptor angiotensin-converting enzyme 2 (ACE2), thereby blocking the virus from attaching to host cells and preventing cellular entry. Elevated levels of serum IgG signify effective defense against viral attacks and control of disease progression (Tegenge et al., 2021). Gruell et al. suggested that decreased serum IgG levels and activity correlate with prolonged COVID-19 infection and reinfection (Gruell et al., 2022). Persistent COVID-19 infection significantly increases the potential risk of developing long COVID, making it crucial to explore factors affecting serum IgG levels to alleviate long COVID (Swank et al., 2023; Davis et al., 2023). Recent studies have reported associations between serum IgG levels and factors such as age, gender, disease severity, and comorbidities in COVID-19 patients (Zeng et al., 2020; Yang et al., 2021; Yazaki et al., 2021; Kutsuna et al., 2021; Toh et al., 2022). However, the number of cases in these studies is relatively small, primarily focusing on severe and critically ill patients. Currently, COVID-19 infections are mainly asymptomatic or mild, and the factors influencing serum IgG levels in these cases remain uncertain.

This study enrolled 1,939 asymptomatic and mild COVID-19 patients hospitalized at Tianjin First Central Hospital from September 2022 to June 2023. It aimed to analyze the relationship between IgG levels, patients’ epidemiological characteristics, and laboratory indicators. The study sought to identify key factors influencing serum IgG levels, assess and intervene early to enhance patients’ immunity to COVID-19, and effectively mitigate the impact of long COVID on their quality of life.

## Materials and methods

### Participants and data source

This study retrospectively collected data on COVID-19 patients hospitalized at Tianjin First Central Hospital from September 2022 to June 2023. The inclusion criteria were defined as follows: (1) confirmed asymptomatic or mild COVID-19 cases (asymptomatic individuals were identified as those who tested positive for SARS-CoV-2 using nucleic acid tests but did not exhibit any related clinical symptoms, such as fever, dry cough, fatigue, sore throat, diminished sense of smell or taste, diarrhea, or other self-reported or clinically detectable signs. Additionally, their CT scans did not show characteristic imaging features of COVID-19. In contrast, mild cases were diagnosed when individuals had a positive nucleic acid test for SARS-CoV-2 and presented with mild clinical symptoms, including low-grade fever, mild fatigue, and disorders of smell and taste, without any imaging evidence of pneumonia), and (2) patients residing locally in Tianjin, China (non-imported cases). The exclusion criteria comprised: (1) cases with incomplete epidemiological information or test results, and (2) patients with acute liver or kidney insufficiency, severe inflammatory diseases, cardiovascular diseases, or immunodeficiency diseases at the time of admission. The diagnosis, clinical classification, and assessment of complications were conducted in accordance with the “COVID-19 Diagnosis and Treatment Protocol (9th Edition)” issued by the National Health Commission of China. Following rigorous screening, a total of 1,939 patients were enrolled in the study, including 1,549 asymptomatic infections and 390 mild cases. All patients provided signed informed consent. This study complies with the Declaration of Helsinki and was approved by the Medical Ethics Committee of Tianjin First Central Hospital (Ethics Committee archiving No. 2022N052KY).

The dataset comprises patients’ epidemiological data, symptoms and signs at admission, nucleic acid test results, and serum levels of antibodies, PCT, and IL-6. These serological measurements were obtained immediately following confirmation of SARS-CoV-2 infection and prior to the administration of any treatment. To ensure data accuracy, the information was obtained directly from the electronic medical records (HIS system) of Tianjin First Central Hospital and independently reviewed by two researchers. Specific IgG and IgM in serum samples were detected using recombinant antigens that specifically target the total SARS-CoV-2 protein via magnetic particle chemiluminescence assay kits (Biology and Science, China). PCT and IL-6 were quantitatively analyzed using electrochemiluminescence immunoassay according to the kit instructions (Ren mai, China). During hospitalization, nasopharyngeal swabs of all patients were tested daily to monitor real-time changes in their condition. To improve test accuracy, each patient’s sample was tested using three different kits (Sheng Xiang, Bo Jie, and Zhi Jiang, China). The Ct value of each sample was calculated according to the kit instructions. The “China Technical Guidelines for Laboratory Testing for COVID-19” sets the Ct threshold for a positive nucleic acid test at 40.

### Statistical analysis

The Shapiro-Wilk method was used to test the normality of the data. Continuous variables with a normal distribution are expressed as mean ± standard deviation, with inter-group differences compared using the t-test. Non-normally distributed continuous variables are expressed as median (upper and lower quartiles), with inter-group differences compared using the Mann-Whitney U test. Categorical variables are expressed as counts (%), and inter-group differences were compared using the chi-square test or Fisher’s exact test. The correlation between two variables was analyzed using Spearman’s tests. Factors affecting patients’ serum IgG levels were predicted using univariate and multivariate logistic regression analyses, and significant predictors were integrated into a nomogram. Finally, ROC curves were plotted, and the AUC calculated to assess the diagnostic performance of the model. All statistical analyses and visualizations were conducted using SPSS 25.0 and R software (version 4.3.2). *P*-values less than 0.05 were considered statistically significant.

## Results

### Clinical characteristics and laboratory examinations of asymptomatic and mild patients

There were 1,939 COVID-19 patients admitted to Tianjin First Central Hospital, including 1,549 asymptomatic cases and 390 mild cases. Among asymptomatic patients, males predominated (51.5%), while among mild cases, females were more prevalent (53.8%). Mild patients had a median age of 37 years (IQR 28-49), slightly older than asymptomatic patients, whose median age was 35 years (IQR 26-48). In laboratory tests, asymptomatic patients showed lower levels of inflammatory markers PCT and IL-6 compared to mild patients, with no statistically significant differences (*P* > 0.05). Asymptomatic patients also had lower serum levels of IgG (median 4.77 g/L, IQR 1.17-14.96 g/L) and IgM (median 0.12 g/L, IQR 0.07-0.24 g/L) compared to mild patients (median 6.41 g/L, IQR 1.93-18.90 g/L for IgG; median 0.14 g/L, IQR 0.08-0.26 g/L for IgM). Interestingly, asymptomatic patients had a significantly shorter time for viral nucleic acid clearance (median 12 days, IQR 9-16 days) than mild patients (median 15 days, IQR 12-18 days), with a statistically significant difference (*P* < 0.05). Additionally, 50.9% of asymptomatic patients received ≥ 3 doses of the COVID-19 vaccine, compared to 45.4% of mild patients, indicating a significant difference (*P* < 0.05). While the proportion of mild patients with comorbidities was higher than that of asymptomatic patients, this difference was not statistically significant. Detailed clinical characteristics and laboratory findings are presented in **Table 1**.

**Table 1:** Baseline clinical and laboratory characteristics of 1,939 asymptomatic or mild COVID-19 cases.

Variables	Asymptomatic patients	Mild patients	*P* value
	(N = 1, 549)	(N = 390)	
Sex			0.058
Male	798 (51.5%)	180 (46.2%)	
Female	751 (48.5%)	210 (53.8%)	
Age			0.976
Median (IQR)	35 (26-48)	37 (28-49)	
IgG (g/L)			**0.037**
Median (IQR)	4.77 (1.17-14.96)	6.41 (1.93-18.90)	
IgM (g/L)			**0.029**
Median (IQR)	0.12 (0.07-0.24)	0.14 (0.08-0.26)	
PCT (ng/mL)			0.876
Median (IQR)	0.04 (0.02-0.07)	0.05 (0.03-0.08)	
IL-6 (U/ml)			0.728
Median (IQR)	10.15 (6.91-15.41)	10.88 (6.99-15.55)	
Days of nucleic acid turn negative			**0.003**
Median (IQR)	12 (9-16)	15 (12-18)	
BMI			0.634
Median (IQR)	24.23 (22.46-26.03)	24.21 (22.26-25.95)	
Vaccine (dose)			**0.048**
<3	760 (49.1%)	213 (54.6%)	
≥3	789 (50.9%)	177 (45.4%)	
Comorbidities			0.562
Hypertension	212 (13.7%)	57 (14.6%)	
Diabetes	216 (13.9%)	62 (15.9%)	
Other	60 (3.9%)	18 (4.6%)	
None	1061 (68.5%)	253 (64.9%)	

Bold values indicate statistical significance.

### Disparities in clinical and laboratory characteristics between patients with IgG < 21.08 g/L and IgG ≥ 21.08 g/L

We analyzed the correlation between serum IgG and other clinical and laboratory indicators (**Table 2**). The results showed that serum IgG was positively correlated with serum IgM and negatively correlated with age, PCT, IL-6, and BMI. Notably, there was a moderate negative correlation between serum IgG and time to nucleic acid negativity (r = -0.578, *P* < 0.001). Based on the mean serum IgG level of 21.08 g/L, patients were divided into two groups: IgG < 21.08 g/L (1,536 patients) and IgG ≥ 21.08 g/L (403 patients). Compared to patients with IgG < 21.08 g/L, those with IgG ≥ 21.08 g/L were younger, had higher serum IgM levels, lower levels of inflammatory markers PCT and IL-6, and a shorter time to nucleic acid negativity. All these differences were statistically significant (*P* < 0.05). Additionally, 46.5% of patients with IgG < 21.08 g/L had received ≥ 3 doses of the COVID-19 vaccine, compared to 62.3% of patients with IgG ≥ 21.08 g/L, indicating a significant difference (*P* < 0.05). The proportion of patients without other comorbidities was significantly higher in the IgG ≥ 21.08 g/L group compared to the IgG < 21.08 g/L group (78.4% vs. 65.0%, *P* < 0.05). More detailed differences in clinical and laboratory characteristics of 1,939 COVID-19 patients grouped by IgG levels are shown in **Table 3**.

**Table 2:** Correlation analysis between serum IgG levels and other clinical and laboratory indicators.

Variables	IgG	
	Correlation coefficient	*P* value
Age	-0.093	0.086
IgM (g/L)	0.171	**0.043**
PCT (ng/mL)	-0.154	0.055
IL-6 (U/ml)	-0.206	**0.029**
Days of nucleic acid turn negative	-0.578	**<0.001**
BMI	-0.035	0.127

Bold values indicate statistical significance.

**Table 3:** Differences in clinical and laboratory characteristics of 1,939 COVID-19 patients grouped by IgG levels.

Variables	IgG<21.08 g/L	IgG≥21.08 g/L	*P* value
	(N = 1,536)	(N = 403)	
Sex			0.154
Male	774 (50.4%)	187 (46.4%)	
Female	762 (49.6%)	216 (53.6%)	
Age			**0.029**
Median (IQR)	36 (27-49)	34 (26-42)	
IgM (g/L)			**0.001**
Median (IQR)	0.12 (0.08-0.22)	0.20 (0.13-0.45)	
PCT (ng/mL)			**0.002**
Median (IQR)	0.05 (0.03-0.08)	0.03 (0.02-0.06)	
IL-6 (U/ml)			**0.005**
Median (IQR)	10.61 (7.15-15.71)	9.09 (5.62-14.56)	
Days of nucleic acid turn negative			**0.006**
Median (IQR)	14 (10-17)	10 (8-15)	
BMI			0.827
Median (IQR)	24.22 (22.34-25.97)	24.18 (22.22-25.95)	
Clinical stage			**0.038**
Asymptomatic	1276 (83.1%)	273 (67.7%)	
Mild	260 (16.9%)	130 (32.3%)	
Vaccine (dose)			**0.002**
<3	821 (53.5%)	152 (37.7%)	
≥3	715 (46.5%)	251 (62.3%)	
Comorbidities			**0.010**
Hypertension	234 (15.2%)	35 (8.7%)	
Diabetes	241 (15.7%)	37 (9.2%)	
Other	63 (4.1%)	15 (3.7%)	
None	998 (65.0%)	316 (78.4%)	

Bold values indicate statistical significance.

### Logistic regression analysis of factors affecting serum IgG levels

**Figure 1:**
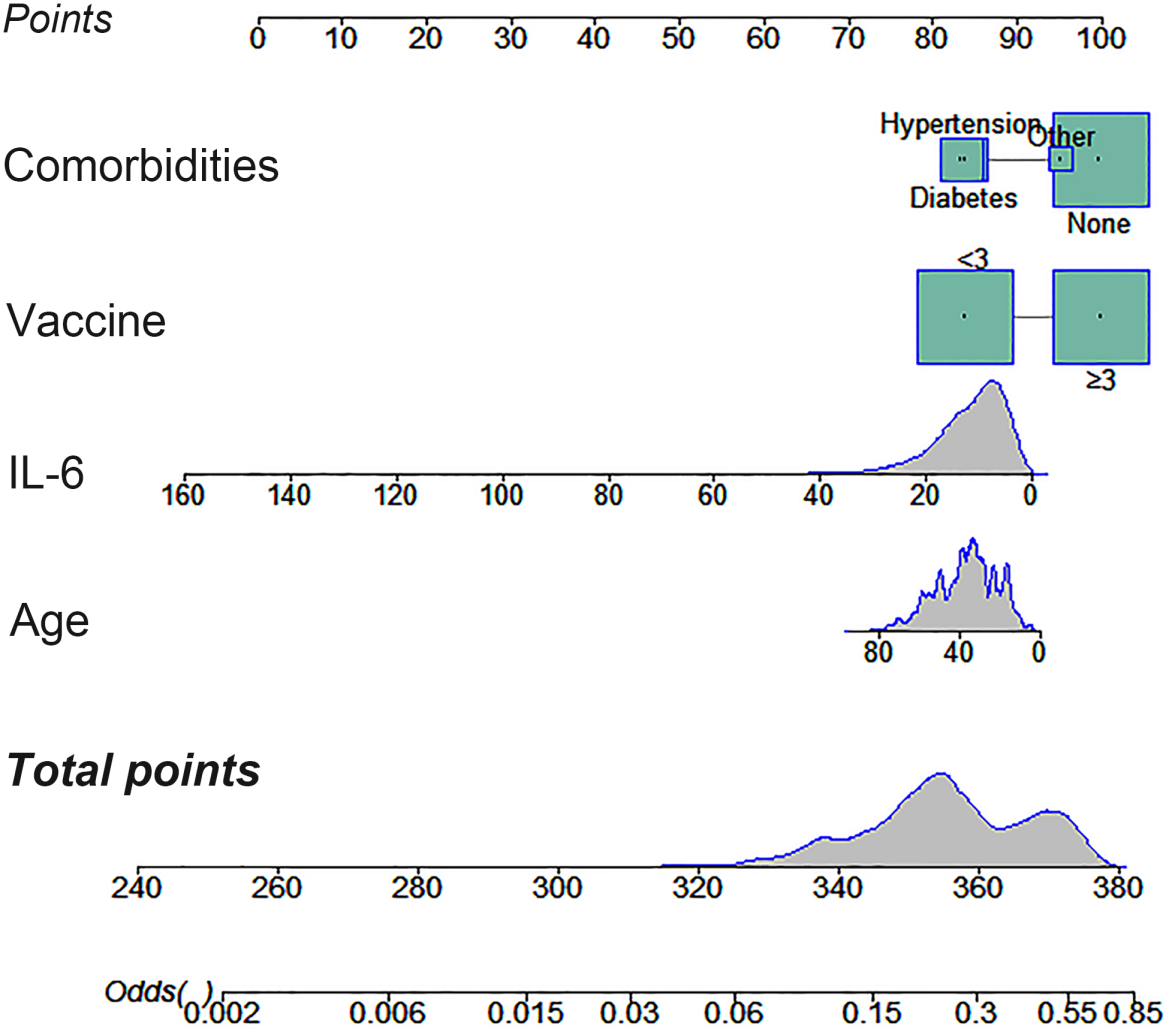
A nomogram constructed to predict patient serum IgG levels based on age, IL-6 levels, COVID-19 vaccination doses, and comorbidities.

**Figure 2:**
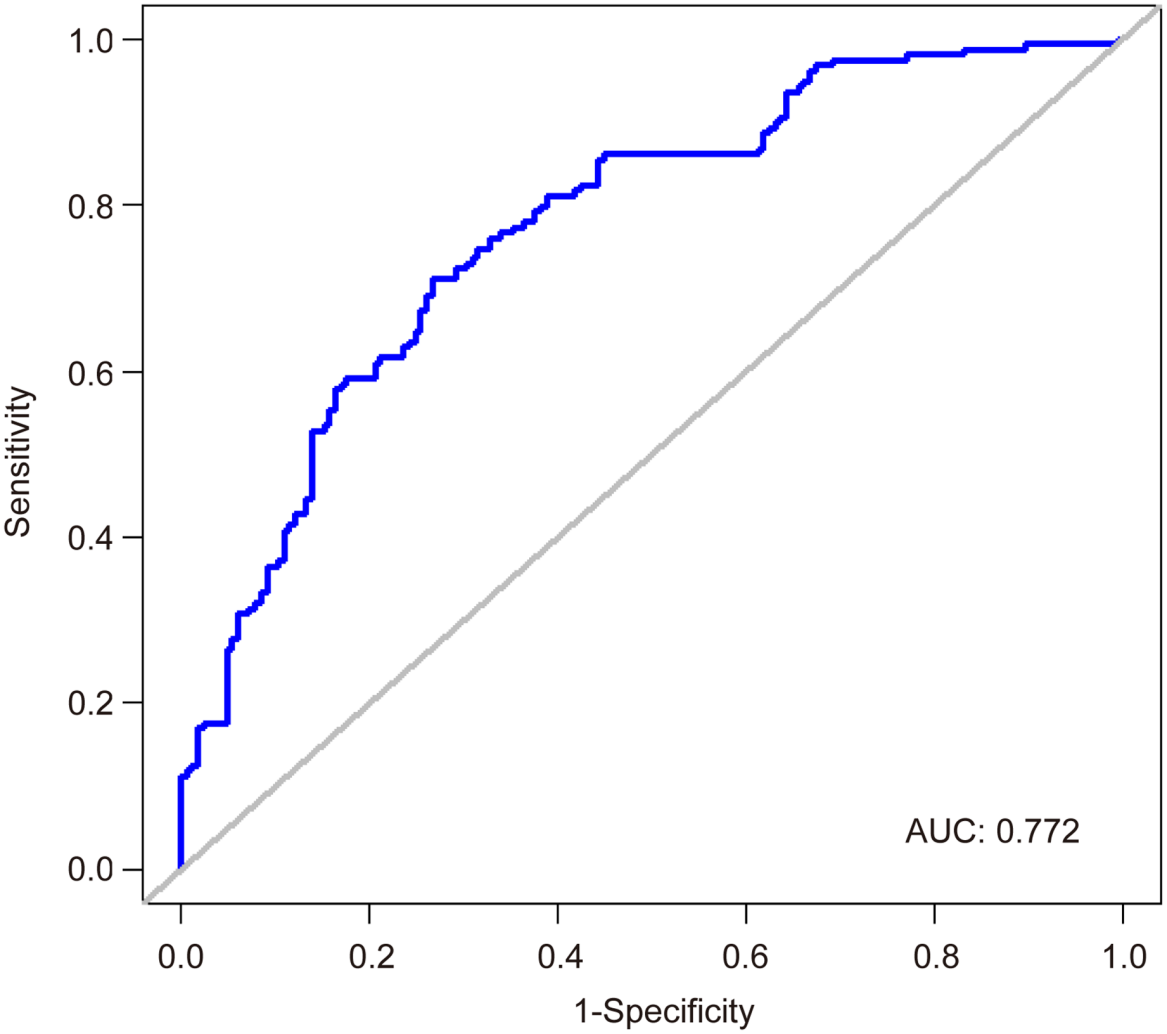
An ROC curve predicting patient serum IgG levels.

To investigate factors influencing serum IgG levels in patients, we initially conducted a univariate logistic regression analysis on all variables. The analysis revealed significant associations between serum IgG levels ≥ 21.08 g/L and younger age, lower IL-6 levels, receiving ≥ 3 doses of COVID-19 vaccine, and absence of comorbidities (*P* < 0.05). Subsequently, these statistically significant variables were included in the multivariate logistic regression analysis. The results demonstrated that age, IL-6 levels, COVID-19 vaccine doses, and comorbidities independently predicted serum IgG levels ≥ 21.08 g/L (**Table 4**, *P* < 0.05). To facilitate visualization, we developed a nomogram for predicting serum IgG levels using age, IL-6 levels, COVID-19 vaccine doses, and comorbidities (**Figure 1**). Each level of the predictive indicators was assigned a score on a rating scale, and the total score was obtained by summing the scores of each predictive factor. Finally, a vertical line was drawn from the total score to the incidence rate axis at the bottom of the nomogram to assess the probability of patients having serum IgG levels ≥ 21.08 g/L. To assess the accuracy of our predictive model, we plotted an ROC curve and calculated an AUC of 0.772 (**Figure 2**).

**Table 4:** Univariate and multivariate logistic regression analyses of factors influencing serum IgG levels.

Variables	Univariate Analysis		Multivariate Analysis		AUC
	HR (95%CI)	*P* value	HR (95%CI)	*P* value	
Sex					
Male	Reference				
Female	1.152 (0.084, 2.221)	0.154			
Age	0.691 (0.684, 0.699)	**0.037**	0.689 (0.681, 0.698)	**0.029**	**0.563**
IgM (g/L)	1.646 (0.950, 2.343)	0.053			
PCT (ng/mL)	0.580(0.136, 1.025)	0.055			
IL-6 (U/ml)	0.969 (0.953, 0.986)	**< 0.001**	0.971 (0.956, 0.987)	**< 0.001**	**0.612**
BMI	0.506 (0.011,1.002)	0.617			
Clinical stage					
Asymptomatic	Reference				
Mild	1.658 (0.923, 2.394)	0.051			
Vaccine (dose)					**0.679**
<3	Reference		Reference		
≥3	1.896 (1.514, 2.279)	**< 0.001**	2.160 (1.707, 2.614)	**< 0.001**	
Comorbidities					**0.538**
None	Reference		Reference		
Hypertension	0.672 (0.524, 0.821)	**0.045**	0.667 (0.518, 0.817)	**0.037**	
Diabetes	0.685 (0.535, 0.836)	**0.042**	0.674 (0.527, 0.822)	**0.034**	
Other	0.852 (0.713, 0.992)	**0.026**	0.804 (0.645, 0.964)	**< 0.001**	
Age+IL-6+Vaccine +Comorbidities					**0.772**

Bold values indicate statistical significance.

## Discussion

As of July 21, 2024, the COVID-19 pandemic has resulted in 775 million infections and 7.05 million deaths worldwide [World Health Organization (WHO). Weekly Epidemiological Update on COVID-19. Available online: https://covid19.who.int/ (accessed on 8 August 2024)]. Upon infection with SARS-CoV-2, the human immune system can become overactivated, releasing large amounts of cytokines, such as tumor necrosis factor-α (TNF-α) and IL-6, triggering an excessive inflammatory response. As COVID-19 transitions into a seasonal coronavirus disease, an increasing number of patients tend to underestimate its health impact. However, ongoing or repeated infections present another serious threat to global health—long COVID, causing long-term impacts on patients’ lungs and various extrapulmonary tissues and organs, including cardiovascular, cognitive, neurological, and metabolic systems (Alkodaymi et al., 2022; Velasquez Garcia et al., 2024; Golzardi et al., 2024; Maniaci et al., 2024). Recent research has discovered that in hospitalized COVID-19 patients, higher antibody levels are correlated with lower inflammation levels (Mink et al., 2024). Thus, early-stage immune function intervention not only aids in halting the virus’s spread within the body but also alleviates the inflammatory response, thereby effectively reducing the negative effects of long COVID on quality of life. In this study, we analyzed the correlation between serum IgG and other variables in 1,939 asymptomatic or mild COVID-19 patients. The results indicated that serum IgG was negatively correlated with age, PCT, IL-6, and BMI, particularly with a moderate negative correlation with nucleic acid clearance time (r = -0.578, *P* < 0.001). To further explore the key factors influencing serum IgG levels in asymptomatic or mild COVID-19 patients, we divided the patients into two groups based on the average serum IgG level of 21.08 g/L: those with IgG < 21.08 g/L and those with IgG ≥ 21.08 g/L. We found that younger age, lower IL-6 levels, receiving ≥ 3 doses of vaccine, and lack of comorbidities were independent predictors of serum IgG levels ≥ 21.08 g/L.

Elderly individuals show reduced peak antibody levels after SARS-CoV-2 infection, likely due to age-related declines in immune function, which diminishes their response to pathogens. Furthermore, viral infections increase the likelihood of severe illness in older adults, thereby slowing down viral clearance (Castilla et al., 2021). Ikezaki et al. observed a significant negative correlation between participants’ anti-spike IgG levels and age (r = -0.35, *P* < 0.01). This correlation remained statistically significant even after adjusting for sex, BMI, smoking or alcohol drinking habits, allergies, and fever or other adverse reactions at the time of vaccination (r = -0.28, *P* < 0.01) (Ikezaki et al., 2022). Similarly, Papaioannidou et al. identified a negative correlation between IgG titers and age (*P* = 0.001). Further stratification of IgG titers by age group revealed significant statistical differences between the youngest (< 30 years) and oldest (> 60 years) groups, as well as between the youngest (< 30 years) and the 51-60 years age group (*P* = 0.001 and *P* = 0.006) (Papaioannidou et al., 2023).

IL-6 is crucial in immune and inflammatory responses during COVID-19 infection. As a pro-inflammatory cytokine, IL-6 promotes leukocyte proliferation and activation through the Janus kinase (JAK)/signal transducer and activator of transcription (STAT) pathway. It drives inflammation by regulating the migration of inflammatory cells and the release of inflammatory mediators (Aletaha et al., 2023). Several studies have shown that IL-6 is closely associated with the onset and severity of COVID-19 (Queiroz et al., 2022; Yin et al., 2023; Wu et al., 2021). Inhibiting IL-6 receptors can effectively reduce COVID-19 complications and mortality (Rubin et al., 2021; Ghosn et al., 2023). Our study found that lower IL-6 levels independently predict serum IgG levels ≥ 21.08 g/L. A possible explanation is that reduced inflammation from lower IL-6 levels creates a stable internal environment, enabling B lymphocytes to secrete more antibodies. Several studies support this correlation. Lu et al. confirmed an inverse correlation between IL-6 expression and SARS-CoV-2 IgG (Lu et al., 2021). In a cross-sectional study by Elhag et al., serum IgM and IgG levels significantly increased (*P* = 0.01, 0.004), while IL-6 and IL-2 levels decreased (*P* = 0.761, 0.071) in discharged patients (Elhag et al., 2023). Masia et al. assessed the impact of IL-6 blockers on long-term immunity in COVID-19 patients and found significantly higher median serum anti-trimeric spike IgG levels (295 vs. 121 BAU/mL; *P* = 0.011) and neutralizing antibodies (74.7 vs. 41.0%IH; *P* = 0.012) within one year post-infection in patients receiving anti-IL-6 therapy. Additionally, a higher proportion of these patients had neutralizing antibodies (80.6% vs. 57.7%; *P* = 0.028) (Masia et al., 2022).

We also found that the absence of comorbidities independently predicts serum IgG levels ≥ 21.08 g/L. This might be because comorbidities induce a chronic inflammatory state, impairing the immune system. Without complications, the body’s internal environment remains stable, allowing B lymphocytes to secrete more antibodies. Guzel et al. found that patients with cardiovascular disease and diabetes had significantly lower serum IgG antibody levels compared to those without these conditions (*P* < 0.05 and *P* < 0.001, respectively) (Guzel et al., 2021). Similarly, Malavazos et al. indicated that 1 to 3 months after the second vaccine dose, patients with abdominal obesity had a more significant reduction in serum anti-trimeric spike IgG levels compared to those without abdominal obesity (2.44-fold [95% CI: 2.22-2.63] vs. 1.82-fold [95% CI: 1.69-1.92], *P* < 0.001) (Malavazos et al., 2022).

To control the spread of the epidemic and reduce severe and fatal cases, large-scale COVID-19 vaccination programs have been repeatedly implemented worldwide. By April 23, 2023, over 13 billion doses of COVID-19 vaccines had been reported to the WHO, offering a 65% to 95% protection rate against COVID-19 (Lin et al., 2023). Our study shows that receiving three or more doses of COVID-19 vaccines independently predicts serum IgG levels ≥ 21.08 g/L, with more doses correlating to higher IgG levels. Notably, it was the most beneficial factor for elevated serum IgG levels. Jiang et al. observed that 28 days after the initial vaccination, the positive rates of serum IgG and IgM were 4% and 13%, respectively. After receiving booster doses, these rates increased to 65% and 97%, respectively (Jiang et al., 2022). Similarly, Wang et al. confirmed that vaccinated patients had significantly higher levels of virus-specific IgG and IgM compared to unvaccinated patients, indicating that COVID-19 vaccination significantly enhances the immune response to SARS-CoV-2 (Wang et al., 2023).

To facilitate clinical application, we developed a nomogram using age, IL-6 levels, COVID-19 vaccination doses, and comorbidities to predict serum IgG levels for the first time. Recognizing that post-treatment serum sample data might obscure the early risk stratification for Long COVID, we collected pretreatment serum samples for antibody, PCT, and IL-6 testing immediately after confirming SARS-CoV-2 infection and before any therapeutic intervention. The developed nomogram offers clinicians a reliable tool for early risk assessment of Long COVID, with an AUC of 0.772, thereby streamlining the treatment decision-making process. Our study suggests that for asymptomatic or mild COVID-19 patients with low IgG levels, using IL-6 blockers, receiving vaccinations, and/or treating comorbidities can enhance the immune response to SARS-CoV-2, thereby reducing the risk of long COVID and improving their quality of life. Despite these insights, the study has its limitations. First, this is a single-center retrospective study, and further multi-center prospective studies are needed to validate our model. Second, the study variables are relatively limited, as we only explored the correlation between a few common laboratory indicators and serum IgG levels. Third, the absence of specific cellular immune response data in our cohort hinders a comprehensive assessment of post-infection immunity and protection. Future research should expand its scope to include more comprehensive laboratory data to refine predictive models.

## Conclusions

In this study, we established a nomogram to predict serum IgG levels in COVID-19 patients. Our results indicate that younger age, lower IL-6 levels, receiving three or more doses of the COVID-19 vaccine, and having no comorbidities are associated with a higher probability of having serum IgG levels ≥ 21.08 g/L. The nomogram we developed demonstrates high clinical predictive performance, making it a reliable tool for estimating the immune response capacity of COVID-19 patients. This aids clinicians in early risk stratification and intervention for long COVID, thereby reducing its negative impact on patients’ quality of life.

## Data Availability

The raw data supporting the conclusions of this article will be made available by the authors, without undue reservation.

## References

[B1] Aletaha D. Kerschbaumer A. Kastrati K. Dejaco C. Dougados M. McInnes I. B.. (2023). Consensus statement on blocking interleukin-6 receptor and interleukin-6 in inflammatory conditions: an update. Ann. Rheumatol. Dis. 82, 773–787. doi: 10.1136/ard-2022-222784 35953263

[B2] Alkodaymi M. S. Omrani O. A. Fawzy N. A. Shaar B. A. Almamlouk R. Riaz M.. (2022). Prevalence of post-acute COVID-19 syndrome symptoms at different follow-up periods: a systematic review and meta-analysis. Clin. Microbiol. Infect. 28, 657–666. doi: 10.1016/j.cmi.2022.01.014 35124265 PMC8812092

[B3] Castilla J. Guevara M. Miqueleiz A. Baigorria F. Ibero-Esparza C. Navascues A.. (2021). Risk factors of infection, hospitalization and death from SARS-coV-2: A population-based cohort study. J. Clin. Med. 10, 1–13. doi: 10.3390/jcm10122608 PMC8231921 34199198

[B4] Davis H. E. McCorkell L. Vogel J. M. Topol E. J. (2023). Long COVID: major findings, mechanisms and recommendations. Nat. Rev. Microbiol. 21, 133–146. doi: 10.1038/s41579-022-00846-2 36639608 PMC9839201

[B5] Elhag W. Elamin B. K. Idris E. Elsheikh A. Ghaleb K. Fallatah I.. (2023). Clinico-epidemiological laboratory findings of COVID- 19 positive patients in a hospital in Saudi Arabia. Infect. Drug Resist. 16, 4845–4856. doi: 10.2147/IDR.S418629 37520449 PMC10386838

[B6] Ghosn L. Assi R. Evrenoglou T. Buckley B. S. Henschke N. Probyn K.. (2023). Interleukin-6 blocking agents for treating COVID-19: a living systematic review. Cochrane. Database. Syst. Rev. 6, CD013881. doi: 10.1002/14651858.CD013881.pub2 37260086 PMC10237088

[B7] Golzardi M. Hromic-Jahjefendic A. Sutkovic J. Aydin O. Unal-Aydin P. Becirevic T.. (2024). The aftermath of COVID-19: exploring the long-term effects on organ systems. Biomedicines 12, 1–21. doi: 10.3390/biomedicines12040913 PMC11048001 38672267

[B8] Gruell H. Vanshylla K. Weber T. Barnes C. O. Kreer C. Klein F. (2022). Antibody-mediated neutralization of SARS-coV-2. Immunity 55, 925–944. doi: 10.1016/j.immuni.2022.05.005 35623355 PMC9118976

[B9] Guzel E. C. Celikkol A. Erdal B. Sedef N. (2021). Immunogenicity after CoronaVac vaccination. Rev. Assoc. Med. Bras. (1992). 67, 1403–1408. doi: 10.1590/1806-9282.20210389 35018966

[B10] Hall V. Foulkes S. Insalata F. Kirwan P. Saei A. Atti A.. (2022). Protection against SARS-CoV-2 after Covid-19 Vaccination and Previous Infection. N. Engl. J. Med. 386, 1207–1220. doi: 10.1056/NEJMoa2118691 35172051 PMC8908850

[B11] Huang C. Huang L. Wang Y. Li X. Ren L. Gu X.. (2023). 6-month consequences of COVID-19 in patients discharged from hospital: a cohort study. Lancet 401, e21–e33. doi: 10.1016/S0140-6736(23)00810-3 37321233 PMC10258565

[B12] Ikezaki H. Nomura H. Shimono N. (2022). Dynamics of anti-Spike IgG antibody level after the second BNT162b2 COVID-19 vaccination in health care workers. J. Infect. Chemother. 28, 802–805. doi: 10.1016/j.jiac.2022.02.024 35288023 PMC8901382

[B13] Jiang R. Dou X. Li M. Wang E. Hu J. Xiong D.. (2022). Dynamic observation of SARS-CoV-2 IgM, IgG, and neutralizing antibodies in the development of population immunity through COVID-19 vaccination. J. Clin. Lab. Anal. 36, e24325. doi: 10.1002/jcla.24325 35235705 PMC8993648

[B14] Kutsuna S. Asai Y. Matsunaga A. Kinoshita N. Terada M. Miyazato Y.. (2021). Factors associated with anti-SARS-CoV-2 IgG antibody production in patients convalescing from COVID-19. J. Infect. Chemother. 27, 808–813. doi: 10.1016/j.jiac.2021.01.006 33531292 PMC7836855

[B15] Lin B. Cheng L. Zhang J. Yang M. Zhang Y. Liu J.. (2023). Immunology of SARS-CoV-2 infection and vaccination. Clin. Chim. Acta 545, 117390. doi: 10.1016/j.cca.2023.117390 37187222 PMC10182659

[B16] Lopez-Leon S. Wegman-Ostrosky T. Perelman C. Sepulveda R. Rebolledo P. Cuapio A.. (2021). More than 50 long-term effects of COVID-19: A systematic review and meta-analysis. Sci. Rep. 11, 16144. doi: 10.1038/s41598-021-95565-8 34373540 PMC8352980

[B17] Lu Q. Zhu Z. Tan C. Zhou H. Hu Y. Shen G.. (2021). Changes of serum IL-10, IL-1beta, IL-6, MCP-1, TNF-alpha, IP-10 and IL-4 in COVID-19 patients. Int. J. Clin. Pract. 75, e14462. doi: 10.1111/ijcp.14462 34107113 PMC8237069

[B18] Malavazos A. E. Basilico S. Iacobellis G. Milani V. Cardani R. Boniardi F.. (2022). Antibody responses to BNT162b2 mRNA vaccine: Infection-naive individuals with abdominal obesity warrant attention. Obes. (Silver. Spring). 30, 606–613. doi: 10.1002/oby.23353 34850576

[B19] Maniaci A. Lavalle S. Masiello E. Lechien J. R. Vaira L. Boscolo-Rizzo P.. (2024). Platelet-rich plasma (PRP) in the treatment of long COVID olfactory disorders: A comprehensive review. Biomedicines 12, 1–17. doi: 10.3390/biomedicines12040808 PMC11048638 38672163

[B20] Masia M. Fernandez-Gonzalez M. Garcia J. A. Padilla S. Garcia-Abellan J. Botella A.. (2022). Robust long-term immunity to SARS-CoV-2 in patients recovered from severe COVID-19 after interleukin-6 blockade. EBioMedicine 82, 104153. doi: 10.1016/j.ebiom.2022.104153 35816896 PMC9265168

[B21] Mink S. Drexel H. Leiherer A. Frick M. Reimann P. Saely C. H.. (2024). Interplay of inflammatory markers and anti-SARS-CoV-2 antibodies in COVID-19 mortality: A prospective cohort study. Int. J. Infect. Dis. 143, 107016. doi: 10.1016/j.ijid.2024.107016 38521446

[B22] Papaioannidou P. Skoumpa K. Bostanitis C. Michailidou M. Stergiopoulou T. Bostanitis I.. (2023). Age, sex and BMI relations with anti-SARS-coV-2-spike igG antibodies after BNT162b2 COVID-19 vaccine in health care workers in northern Greece. Microorganisms 11, 1–13. doi: 10.3390/microorganisms11051279 PMC10220759 37317253

[B23] Queiroz M. A. F. Neves P. Lima S. S. Lopes J. D. C. Torres M. Vallinoto I.. (2022). Cytokine profiles associated with acute COVID-19 and long COVID-19 syndrome. Front. Cell. Infect. Microbiol. 12. doi: 10.3389/fcimb.2022.922422 PMC9279918 35846757

[B24] Rubin E. J. Longo D. L. Baden L. R. (2021). Interleukin-6 receptor inhibition in covid-19 - cooling the inflammatory soup. N. Engl. J. Med. 384, 1564–1565. doi: 10.1056/NEJMe2103108 33631064 PMC7944949

[B25] Soheili M. Khateri S. Moradpour F. Mohammadzedeh P. Zareie M. Mortazavi S. M. M.. (2023). The efficacy and effectiveness of COVID-19 vaccines around the world: a mini-review and meta-analysis. Ann. Clin. Microbiol. Antimicrob. 22, 42. doi: 10.1186/s12941-023-00594-y 37208749 PMC10198032

[B26] Soriano J. B. Murthy S. Marshall J. C. Relan P. Diaz J. V. Condition, W. H. O. C. C. D. W. G. o. P.-C.- (2022). A clinical case definition of post-COVID-19 condition by a Delphi consensus. Lancet Infect. Dis. 22, e102–e107. doi: 10.1016/S1473-3099(21)00703-9 34951953 PMC8691845

[B27] Swank Z. Senussi Y. Manickas-Hill Z. Yu X. G. Li J. Z. Alter G.. (2023). Persistent circulating severe acute respiratory syndrome coronavirus 2 spike is associated with post-acute coronavirus disease 2019 sequelae. Clin. Infect. Dis. 76, e487–e490. doi: 10.1093/cid/ciac722 36052466 PMC10169416

[B28] Tegenge M. A. Mahmood I. Struble E. Golding B. (2021). Dosing considerations for antibodies against COVID-19. Drugs R. D. 21, 1–8. doi: 10.1007/s40268-020-00330-3 33259037 PMC7705402

[B29] Toh Z. Q. Anderson J. Mazarakis N. Neeland M. Higgins R. A. Rautenbacher K.. (2022). Comparison of seroconversion in children and adults with mild COVID-19. JAMA. Netw. Open 5, e221313. doi: 10.1001/jamanetworkopen.2022.1313 35262717 PMC8908077

[B30] Trobajo-Sanmartin C. Miqueleiz A. Guevara M. Fernandez-Huerta M. Burgui C. Casado I.. (2023). Comparison of the risk of hospitalization and severe disease among co-circulating severe acute respiratory syndrome coronavirus 2 variants. J. Infect. Dis. 227, 332–338. doi: 10.1093/infdis/jiac385 36179126

[B31] Velasquez Garcia H. A. Wong S. Jeong D. Binka M. Naveed Z. Wilton J.. (2024). Risk of major adverse cardiovascular events after SARS-coV-2 infection in british columbia: A population-based study. Am. J. Med. 1–8. doi: 10.1016/j.amjmed.2024.04.010 38670520

[B32] Wang J. Dong H. Zhao J. Li T. Wang M. Zhou C.. (2023). Effects of vaccines on clinical characteristics of convalescent adult patients infected with SARS-CoV-2 Omicron variant: A retrospective study. Front. Microbiol. 14. doi: 10.3389/fmicb.2023.1096022 PMC10101175 37065120

[B33] Wu J. Shen J. Han Y. Qiao Q. Dai W. He B.. (2021). Upregulated IL-6 indicates a poor COVID-19 prognosis: A call for tocilizumab and convalescent plasma treatment. Front. Immunol. 12. doi: 10.3389/fimmu.2021.598799 PMC7969719 33746945

[B34] Yang H. S. Costa V. Racine-Brzostek S. E. Acker K. P. Yee J. Chen Z.. (2021). Association of age with SARS-coV-2 antibody response. JAMA. Netw. Open 4, e214302. doi: 10.1001/jamanetworkopen.2021.4302 33749770 PMC7985726

[B35] Yazaki S. Yoshida T. Kojima Y. Yagishita S. Nakahama H. Okinaka K.. (2021). Difference in SARS-coV-2 antibody status between patients with cancer and health care workers during the COVID-19 pandemic in Japan. JAMA. Oncol. 7, 1141–1148. doi: 10.1001/jamaoncol.2021.2159 34047762 PMC8164151

[B36] Yin J. X. Agbana Y. L. Sun Z. S. Fei S. W. Zhao H. Q. Zhou X. N.. (2023). Increased interleukin-6 is associated with long COVID-19: a systematic review and meta-analysis. Infect. Dis. Poverty. 12, 43. doi: 10.1186/s40249-023-01086-z 37095536 PMC10123579

[B37] Zeng F. Dai C. Cai P. Wang J. Xu L. Li J.. (2020). A comparison study of SARS-CoV-2 IgG antibody between male and female COVID-19 patients: A possible reason underlying different outcome between sex. J. Med. Virol. 92, 2050–2054. doi: 10.1002/jmv.25989 32383183 PMC7267228

